# Plasmocytome cutané secondaire révélant un myélome multiple: à propos d'un cas

**DOI:** 10.11604/pamj.2016.24.44.8697

**Published:** 2016-05-10

**Authors:** Lydie Ocini Ngolet, Norbert Lamini N'soundhat, Eliane Ndounga, Innocent Kocko, Daphtone Chabel Nkouala Kidédé, Honoré Ntsiba

**Affiliations:** 1 Service d'Hématologie Clinique, CHU de Brazzaville, Congo; 2 Service de Rhumatologie, CHU de Brazzaville, Congo; 3 Service de Carcinologie, CHU de Brazzaville, Congo

**Keywords:** Plasmocytome cutané, myélome, Afrique, Cutaneous plasmacytoma, myeloma, Africa

## Abstract

Le plasmocytome cutané secondaire métastatique est une prolifération plasmocytaire multiple extramédullaire de localisation cutanée. Son diagnostic repose sur la mise en évidence d'une prolifération plasmocytaire maligne au niveau médullaire et cutané. Son apparition s'associe à un stade avancé du myélome et à un pronostic péjoratif.

## Abstract (en)

Secondary metastatic cutaneous plasmacytoma is a multiple extramedullary plasma cell proliferation involving skin. Its diagnosis is based on the identification of malignant plasma cells proliferation in the bone marrow and in the skin. Its occurrence is associated with advanced myeloma and a poor prognosis.

## Introduction

Les plasmocytomes cutanés sont extrêmement rares et représentent moins de 2% des plasmocytomes extramédullaires [1]. Ils sont répartis en deux catégories selon qu'ils sont associés ou pas à un myélome, en plasmocytome primitif ou secondaire. Nous rapportons la première observation Africaine d'un plasmocytome secondaire métastatique cutané chez une patiente de 71 ans révélant un myélome.

## Patient et observation

**Figure 1:**
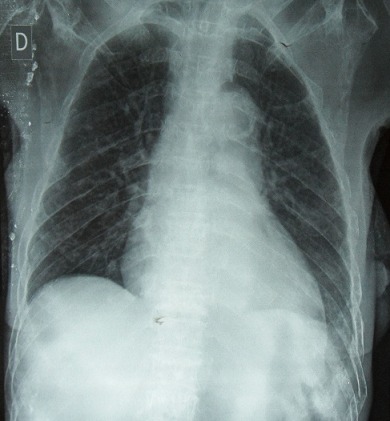
Radiographie thoracique de face montrant des fractures multi-étagées de la 6^ème^, 7^ème^ et 8^ème^ côte droite

**Figure 2:**
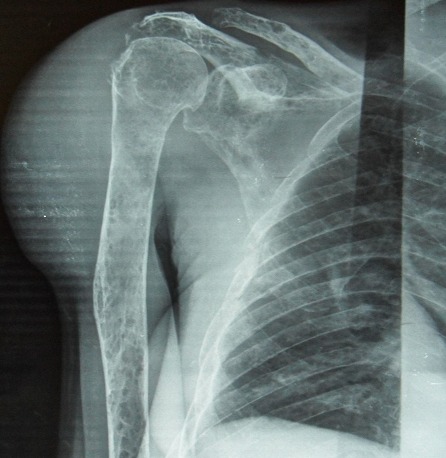
Radiographie de face de l'humérus droit montrant une tuméfaction en regard de l’épiphyse et diaphyse humérale correspondant à un plasmocytome cutané: déminéralisation diffuse avec un amincissement des corticales; lésions ostéolytiques multiples à type de geode

**Figure 3:**
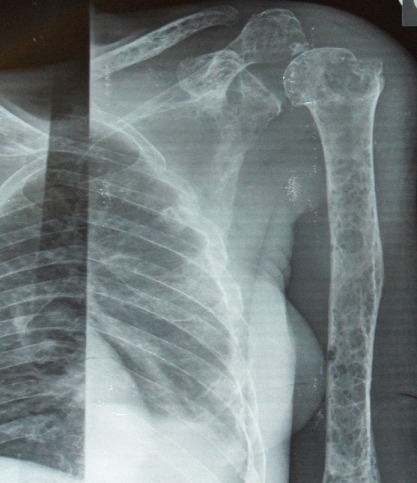
Radiographie de l'humérus gauche mettant en évidence une déminéralisation humérale diffuse, un amincissement des corticales et des géodes à l'emporte-pièce diaphysaire

Une patiente de 71 ans, originaire du Congo, est admise dans le service de Rhumatologie du Centre Hospitalier de Brazzaville au Congo pour l'exploration de tuméfactions cutanées douloureuses des deux poignets, des épaules, des genoux et du thorax évoluant depuis trois ans. Les douleurs étaient invalidantes et de rythme inflammatoire. Ce tableau était associé à un amaigrissement progressif. La patiente présentait une altération de l’état général avec un score EGOG à 3. L'examen des 2 mains, épaules, genoux et de la face antérieur du thorax montrait des tuméfactions cutanés en regard des articulations, en relief, arrondies, molles, de 4-6 cm de diamètre sans augmentation de la chaleur locale, ni fluctuance. Il y a une réduction de l'amplitude des articulations inter phalangiennes, des genoux et de l’épaule gauche. Un syndrome osseux diffus axial et périphérique était noté. Les examens biologiques ont noté une anémie normocytaire, normochorme à 10.2 g/dL, une altération de la fonction rénale avec une créatininémie à 361umol/L, une uricémie à 124 mg/L, une hypercalcémie à 122mg/L. L’électrophorèse des protides a montré un pic monoclonal en position gamma avec un taux de 27g/L, une hypoalbuminémie à 41 g/L. L'immunoélectrophorèse a retrouvé une immunoglobuline monoclonale de type IgG à 32g/L. La recherche des facteurs rhumatoïdes était négative. L'examen histologique de la biopsie cutanée décrit un infiltrat plasmocytaire dermique en nappe sans atteinte du muscle strié en faveur d'un plasmocytome de localisation cutané. Le myélogramme a montré une prolifération plasmocytaire maligne à 38% confirmant ainsi le diagnostic du myélome multiple. Le bilan radiologique montre: une fracture de la 6^ème^,7^ème^ et 8^ème^ côte droite (Figure 1), un épaississement des parties molles en regard de la tête et 1/3 supérieur de la tête humérale droite, une déminéralisation des diaphyses humérales diffuses avec amincissement des corticales, des géodes à l'emporte-pièce du crâne et des diaphyses humérales (Figure 2 et Figure 3). Nous avons retenu le diagnostic de plasmocytomes cutanés métastatiques associés à un myélome stade IIIB selon la classification de Salmon et Durie. Devant l'hypercalcémie, nous avons instauré en urgence une hyperhydratation, diurèse forcée et bolus de corticoïdes, ce durant 4 jours suivi d'une polychimiothérapie de type MD (melphalan, déxaméthasone). La patiente est décédée au troisième jour de chimiothérapie.

## Discussion

Les plasmocytomes extra-médullaires sont des tumeurs situées en dehors de la moelle osseuse. Ils sont décrits comme associés au myélome dans 70% de cas d'autopsiés [1]. Les localisations extra osseuses se font essentiellement au dépend des voies aériennes supérieures et du tube digestif [2–4]. Les plasmocytomes de localisation cutanée sont extrêmement rares [1, 2, 4]. Ils doivent être distingués des lymphomes cutanés [5]. Les Plasmocytomes secondaires cutanés (PSC) se distinguent des plasmocytomes primaires cutanés (PCP) par leur association au myélome [2]. Ils sont dit primaires lorsqu'ils sont isolés et secondaires, lorsqu'ils sont associés ou en contigüité avec des lésions osseuses résultant d'un myélome. Les PSC sont dit métastatiques (PSCM) lorsqu'il existe plusieurs lésions cutanées. Patterson et al, distinguent deux types de lésions cutanées dans les plasmocytomes cutanés. Les lésions infiltratives observées uniquement chez les sujets atteints de PSC, et les lésions papillaires voir nodulaires rencontrées voir dans les formes primaires et secondaires [6]. Les PCS sont souvent associés à une prolifération monoclonale IgG dans 56% des cas [7]. Les PSC apparaissent dans l’évolution d'un myélome à forte masse tumorale et s'associent à une évolution rapidement fatale. Patterson et al sur une série de trois cas, rapportèrent une durée de survie de 3 semaines après l'apparition de PSC [6]. Dans notre cas, les lésions cutanés sont rapportées comme évolutives depuis prés de trois ans. Nous n'avons pu déterminer avec certitude qui du myélome ou du plasmocytome était apparu avant. Cependant, la présence au moment du diagnostic d'une prolifération plasmocytaire maligne médullaire et cutané, d'une sécrétion monoclonale d'immunoglobuline, et la présence de lésions osseuses lytiques, sont autant d’éléments qui nous ont permis de poser le diagnostic de PCSM associés à un myélome. Il n'existe aucun traitement codifié du PSC. Il associe en règle générale une chimiothérapie associée à une radiothérapie.

## Conclusion

Le plasmocytome cutané secondaire est une manifestation du stade avancé du myélome multiple.
